# Sparse deconvolution of high-density super-resolution images

**DOI:** 10.1038/srep21413

**Published:** 2016-02-25

**Authors:** Siewert Hugelier, Johan J. de Rooi, Romain Bernex, Sam Duwé, Olivier Devos, Michel Sliwa, Peter Dedecker, Paul H. C. Eilers, Cyril Ruckebusch

**Affiliations:** 1 Université de Lille, LASIR CNRS UMR 8516, F-59000 Lille, France; 2 Erasmus MC, Department of Biostatistics, Rotterdam, the Netherlands; 3 Department of Chemistry, KU Leuven, Belgium; 4 Swammerdam Institute for Life Sciences (Universiteit van Amsterdam), 1098 XH Amsterdam, The Netherlands

## Abstract

In wide-field super-resolution microscopy, investigating the nanoscale structure of cellular processes, and resolving fast dynamics and morphological changes in cells requires algorithms capable of working with a high-density of emissive fluorophores. Current deconvolution algorithms estimate fluorophore density by using representations of the signal that promote sparsity of the super-resolution images via an L_1_-norm penalty. This penalty imposes a restriction on the sum of absolute values of the estimates of emitter brightness. By implementing an L_0_-norm penalty – on the number of fluorophores rather than on their overall brightness – we present a penalized regression approach that can work at high-density and allows fast super-resolution imaging. We validated our approach on simulated images with densities up to 15 emitters per μm^-2^ and investigated total internal reflection fluorescence (TIRF) data of mitochondria in a HEK293-T cell labeled with DAKAP-Dronpa. We demonstrated super-resolution imaging of the dynamics with a resolution down to 55 nm and a 0.5 s time sampling.

Super-resolution wide-field fluorescence microscopy can provide structural information at the nanoscale and dynamic insight about biological processes in live or fixed cell sampless^1^,^2^. In general, the available information in super-resolution images is related to the density of the emitters, with more emitters leading to more information, and to their localization uncertainty^3^,^4^. One of the strategies for obtaining a high spatial resolution is based on the sequential imaging and localization of sparse subsets of blinking fluorophores distributed over thousands of frames, resulting in a high-density image of their positions and intensities^5^,^6^,^7^. However, to obtain a high spatial resolution on short time sampling and potentially probe dynamic processes in live cells, this principle must be extended to the analysis of high-density frames^8^,^9^,^10^,^11^,^12^ in which many emitters are simultaneously active and the emissions strongly overlap. Since single-emitter fitting methods collapse when applied to high-density super-resolution data, the development of new image processing algorithms remains a topical and challenging issue for dynamic imaging and faster super-resolution experiments^11^,^13^,^14^,^15^,^16^.

The cornerstone of high-density localization is the combination of computer reconstruction of the image with additional regularization by imposing sparsity of the solution, i.e. setting an indirect limitation on the number of emitters that can be present. This is the approach taken by deconvolution methods^14^,^15^ that estimate the density of fluorophores for each frame independently. These methods have shown better performance in situations where the labeling density is more than ten times higher than what can be analyzed by single-emitter fitting algorithms. Some limitations however remain, mainly related to the fact that these methods do not estimate distinct emitter positions but instead sample the overall emitter density and brightness on a sub-pixel grid. Here, we present a new deconvolution approach encoding sparsity with a penalty on the number of emitters. When monitoring protein-labeled mitochondria in a HEK293-T cell, we show that our method can work with higher densities of active emitters and produce high-fidelity super-resolution images from the analysis of only a few frames. It illustrates that our approach can be beneficial for faster resolution imaging in order to resolve fast dynamics, or to reduce movement artifacts.

Deconvolution methods in super-resolution microscopy find solutions of the ill-conditioned inverse problem ***y** = **Cx***where the elements in the signal vector ***x*** are the intensities of the fluorophores, discretized on a sub-pixel grid finer than the pixelated raw image, the vector ***y*** corresponds to the observed camera image and ***C*** is a convolution matrix defined by the point spread function (PSF) of the instrument. Provided the sub-pixel grid has a sufficiently fine spacing, an assumption of sparsity can be imposed on the signal vector***x***as the number of active fluorophores is limited. This allows their positions to be recovered even for high-density frames (i.e. images where there is a high degree of overlap in the emission of the fluorophores). The results obtained should reflect the prior knowledge that the specimen is labeled with discrete fluorophores and the local density should be everywhere zero except at the position of fluorophores. By running the algorithm for each high-density frame and merging the results, a super-resolution image can then be reconstructed. State-of-the-art high-density methods^14^,^15^,^16^ adopt techniques promoting sparse representation of the super-resolution images based on an L_1_-norm penalty. This penalty imposes a restriction on the sum of the absolute values of the elements in ***x***^17^,^18^, i.e. on the sum of absolute values of emitter brightness. Despite its practical usefulness to induce sparsity of the images, there are some limitations associated to the use of an L_1_-norm, such as spatial bias and underestimation of the photon counts. To overcome these issues, one alternative is to combine deconvolution with an L_1_-norm penalty and continuous localization in a region of interest for the refinement of the positions of the fluorophores^16^. However, an L_1_-norm penalty does not strictly translate the real properties of single-molecule fluorescence signals. Fluorescence intensities of individual emitters vary strongly based on their local environment, or their orientation, and, as a result, there is little physical basis for imposing penalties on the overall fluorophore brightness. A much more relevant approach is to impose a penalty on the number of fluorophores per image (or frame). This can be achieved with an L_0_-norm penalty that is constraining the total number of non-zero elements in ***x***, whatever their intensities. This penalty matches the properties of the signals emitted by point-like fluorophores better, and can produce better quantitative results when estimating the emitter density, as illustrated in Fig. 1. Here, an image that consists of 20 emitters was simulated (see Fig. 1a). In Fig. 1b, the solution obtained with an L_1_-norm penalty required 66 emitters whereas, in Fig. 1c, by imposing an L_0_-norm penalty, the number of estimated emitters was limited to 21. Although the two approaches reproduce the simulated image equally well, it should be noted that a much sparser solution was obtained with an L_0_-norm and individual emitters with good intensity estimations could be found.

## Results and Discussion

**Figure 1:**
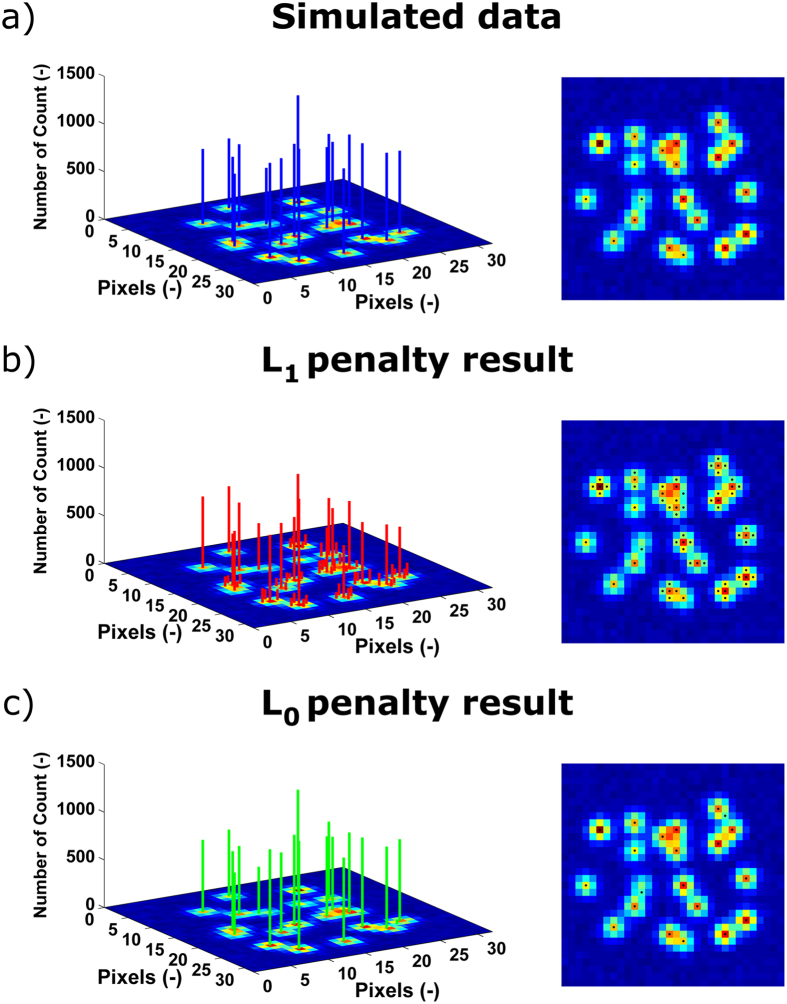
Comparison between different approaches for penalized least squares regression. Fluorophore positions and intensities are indicated by the vertical lines. Fluorophore positions are also shown in top view in the images on the right; (**a**) Simulated image consisting of 20 overlapping emitters, corresponding to a density of 1.25 μm^−2^ (see **Methods** section); (**b**) Image reconstruction based on an L_1_-norm penalty and (**c**) based on an L_0_-norm penalty. The images in (**b**,**c**) correspond to the sparsest solutions that could be obtained under the constraint to reconstruct the simulated image with a recall rate of 100%.

The example provided in Fig. 1 emphasizes that minimizing an L_1_-norm in a regression context distorts resolution, as this penalty induces shrinkage of the magnitude of the active coefficients, to which stray emitters (i.e. emitters with a low intensity) get associated. In contrast, for an L_0_-norm penalty, the intensity of the individual emitters recovered is in agreement with the ones of the simulations. We provide in Supplementary Table S1 comprehensive results that translate that images obtained with the L_0_-norm penalty are more quantitative as they estimate the number of true emitters and their intensity more closely. It should be noted that for the results presented in Fig. 1 and Supplementary Table S1, the L_0_- and L_1_-norm penalty parameter were chosen so that the solutions are the sparsest ones that allow full reconstruction (recall rate of 100%) of the simulated image. Starting from there, we designed an algorithm for SParse Image DEconvolution and Reconstruction (SPIDER), which tackles the deconvolution problem in a penalized regression framework by implementing an approximation of the L_0_-norm^19^. We refer to the **Methods** section for a full explanation of the method and to Supplementary Notes and Supplementary Table S2 for more details on the computational strategies.

**Figure 2:**
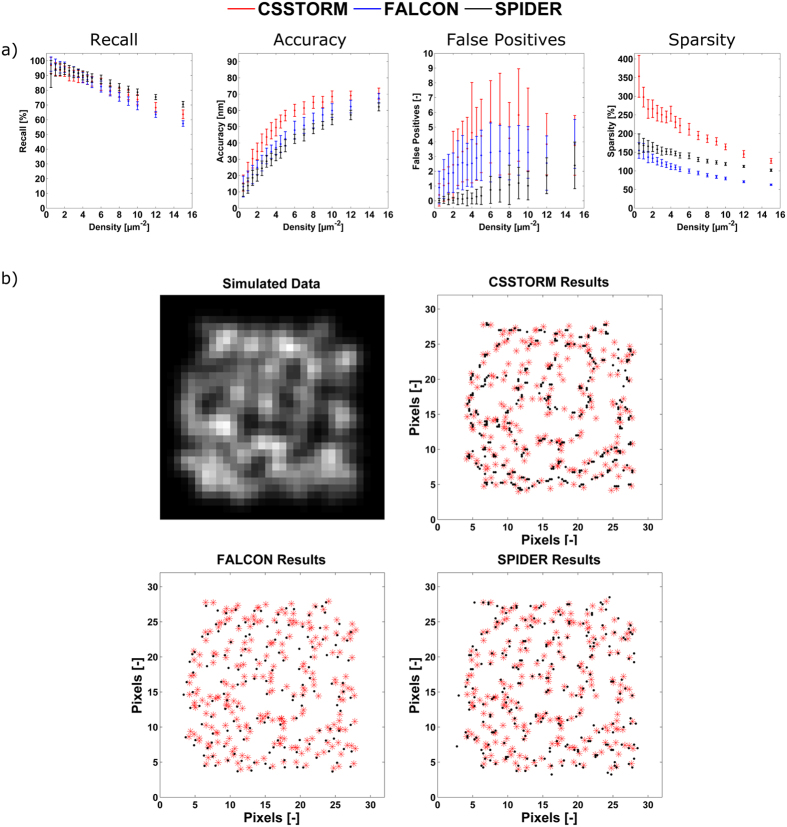
Performance benchmark. (**a**) Figures of merit for CSSSTORM (red), FALCON (blue) and SPIDER (black). The simulation images correspond to an average photon number of 5,000 per molecule (standard deviation of 2,000) and a background of 100 photons with a Poisson distribution (see **Methods** section); the width of the PSF of the emitters is 390 nm and pixels have a size of 166 nm. Calculations were repeated in 50-fold; (**b**) Comparison of the reliability of CSSTORM, FALCON and SPIDER for a simulation map of randomly placed emitters with a density 15 μm^−2^ (benchmark results shown in Fig. 2a), corresponding to 240 molecules on a 24 by 24 pixel map (red crosses and black dots mark true and estimated positions, respectively).

Simulation images (with a density ranging from 0.5–15 μm^−2^), in which the results of the analysis are compared with known positions of the simulated fluorophores, were then used to evaluate the performance of the proposed algorithm. To statistically evaluate the performance of the method, every density was investigated in 50-fold. The **Methods** section explains more about the signal modeling and the exact conditions of the simulations. We first analyzed simulated images for emitters with a width of the PSF of 390 nm on a map with pixels of 166 nm. In Fig. 2, the results obtained with SPIDER are compared to the ones obtained with CSSTORM^14^ and FALCON^16^, which are two methods that impose a sparsity prior on the distribution of fluorophores based on an L_1_-norm. Both methods have demonstrated the capability to identify molecules at high-density. However, FALCON implements an additional continuous refinement of the fluorophore positions whereas SPIDER and CSSTORM provide high-resolution density images on a super-resolution grid (here with a pixel size corresponding to 33 nm). For these latter, the results were thus converted into spatial coordinates to enable method comparison. The approach taken for the evaluation is detailed in the **Methods** section and Supplementary Fig. S1. Recall rate and accuracy are provided and both have a direct impact on the quality of the reconstructed image. However, on their own, they do not allow assessing a comprehensive and reliable evaluation of the overall performance of the algorithm. Therefore, we also report the number of false positives and the sparsity of the solution (i.e. the ratio of the number of non-zero pixels to the number of true fluorophores). At lower densities, the results obtained by the three algorithms do not show significant difference (significance threshold was set at 0.05). However, for image densities larger than 6 μm^−2^, SPIDER outperforms CSSTORM and FALCON in terms of recall rate and accuracy, and a much lower number of false positives is obtained. Moreover, SPIDER also returns sparser images than CSSTORM whereas the solutions provided by FALCON are, in contrast, too sparse at high-density. It could be argued that for the investigated densities, a random placement of the emitters provides good results for the different figures of merit. Therefore, we report In Supplementary Table S3 the results obtained for random distributions of emitters. For solutions corresponding to a density of 15 μm^−2^, a recall of 52.95 ± 7.82% was obtained, which, compared to the results obtained for CSSTORM, FALCON and SPIDER (63.70 ± 2.95%, 56.68 ± 1.79% and 70.48 ± 1.89%, respectively), clarifies that the before mentioned methods contribute to correctly restoring the super-resolution image, even at high densities.

In Fig. 2b, we show high-density simulated images in which we demonstrate the robustness of the SPIDER results on a random distribution of fluorophores with a density of 15 μm^−2^. From this figure, it should be clear that the SPIDER image provides a more quantitative and unbiased interpretation compared to CSSTORM and FALCON, where the obtained images are either too dense (and interpretation could suggest the presence of a false structure) or too sparse (with loss of information), respectively. In addition, we provide in Supplementary Fig. S2 the results obtained for simulated images corresponding to a larger ratio of the PSF to pixel size, i.e. a width of the PSF of 390 nm with a pixel size of 100 nm, more favorable to FALCON.

**Figure 3:**
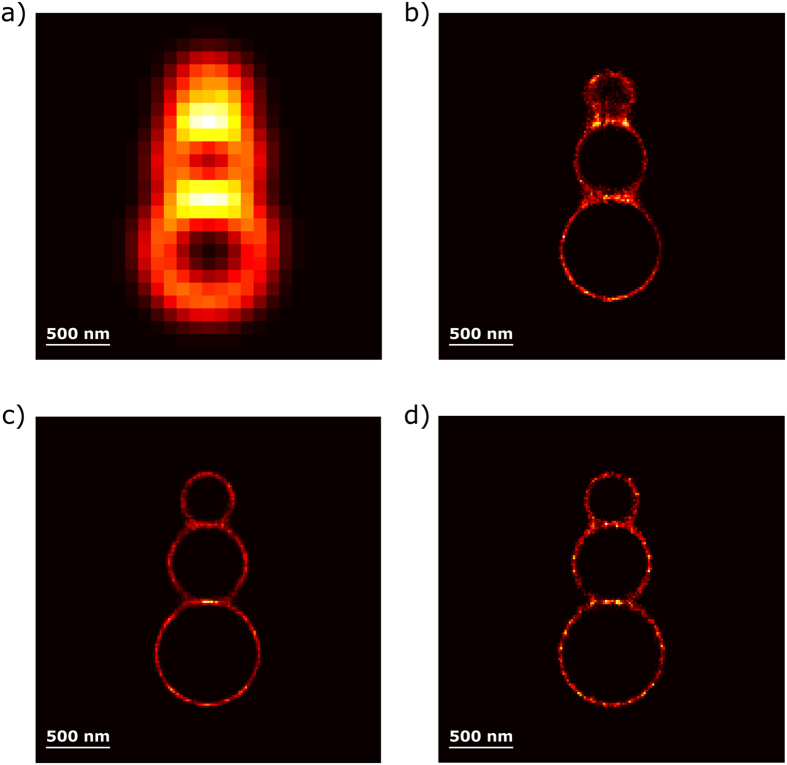
Results obtained for a series of three juxtaposed circles with a diameter of 400, 600 and 800 nm, respectively. The emitters are positioned every 20 nm on the circles with a τ_off_/τ_on_ ratio of 31, leading to an average of 18 active fluorophores per frame (fluorophore density of 15 μm^−2^). We report in (**a**) the average image over 300 frames of the simulated data and the results of (**b**) CSSTORM; (**c**) FALCON and (**d**) SPIDER. The results show that CSSTORM show some spatial distortion in the position of the emitters, while FALCON and SPIDER allow the structure to be recovered reliably (for more details, see Supplementary Fig. S3).

Figure 3 shows the results for high-density simulated data of a circular geometric structure. The three juxtaposed circles with a diameter of 400, 600 and 800 nm contain emitters positioned 20 nm from one another (see **Methods** section). As observed, SPIDER allows the entire structure to be reliably recovered; similar to the localization positions obtained by FALCON, whereas CSSTORM introduces some spatial distortion in emitter positions and in estimation of the circles diameters (see Supplementary Fig. S3), as previously reported in reference^16^. It should be noted that the SPIDER and CSSTORM images are expressed on a sub-pixel grid of 20 nm while the FALCON image is localized in a continuous space. Overall, only SPIDER works well under the two regimes evaluated here (random distribution of the fluorophores and geometrically distributed fluorophores).

**Figure 4:**
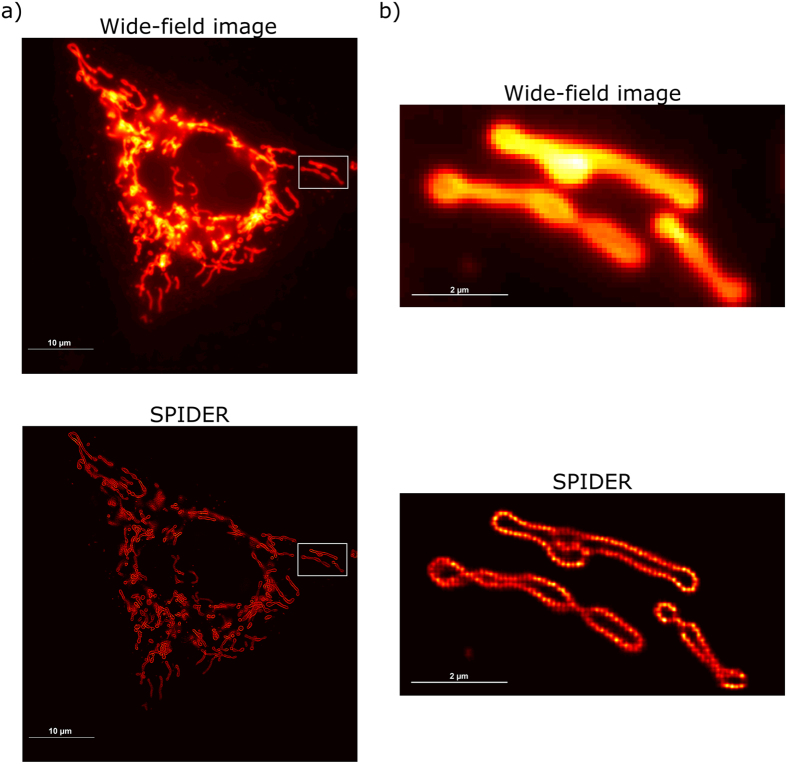
Mitochondria in a HEK293-T cell labeled with DAKAP-Dronpa visualized in Epi-illumination mode. Experiments were performed on an Olympus cellTIRF microscope equipped with an additional EMCCD camera. Images (1,000 frames) were recorded with a constant 488 nm laser excitation and a camera exposure time of 30 ms: (**a**) Average live-cell image and SPIDER reconstruction of the hollow mitochondria structures (averaging of density estimations over 1,000 frames); (**b**) Average live-cell image and SPIDER reconstruction (averaging of density estimations over 1,000 frames) of the region indicated by the box in (**a**), sized 4 μm by 8 μm. Note the blurred and thickened membranes which indicate movement of the cell during acquisition (see Supplementary Fig. S4).

Lastly, we validated the performance of the proposed approach by applying SPIDER to a series of total internal reflection fluorescence (TIRF) microscopy images of mitochondria in a HEK293-T cell labeled with DAKAP-Dronpa. A video of the raw acquisition is supplied in Supplementary Video S1. It contains 1,000 frames acquired over 30 s in which blinking can hardly be detected, in comparison with typical low density super-resolution data. Our results in Fig. 4 correspond to a super-resolution grid with a pixel size of 25 nm and show dramatic improvement in resolution compared to the mean image of the wide-field data (Fig. 4b shows the zoomed region, indicated by the box in Fig. 4a). Note that bright areas of the wide-field image translate into areas with a high density on the SPIDER image (the SPIDER image was rendered using a PSF of 25 nm). The zoomed region in Fig. 4b illustrates that hollow morphologies can be resolved. This morphology is plausible given the fact that the construct used here incorporates a targeting motif from DAKAP-1^20^, which targets the outer membrane of the mitochondria. However, the data were obtained with protein labels expressed at very high levels, which may impair biological function. In addition, we provide in supporting information a movie (Supplementary Video S2) showing the individual positions spotted by SPIDER over 3 s (i.e. 100 frames).

**Figure 5:**
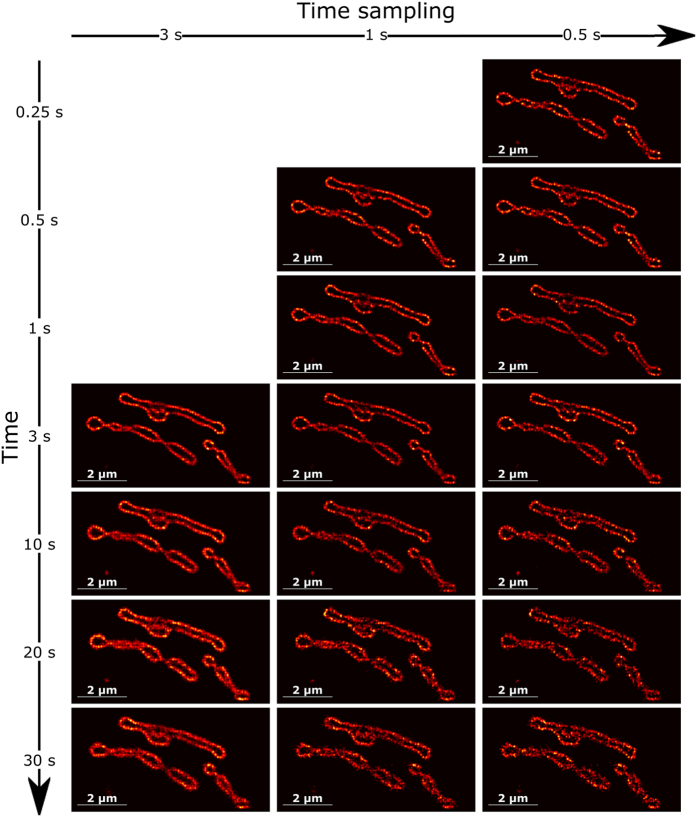
SPIDER reconstruction of the region indicated by the box in Fig. 4a. The different images are reconstructed at different times and for a time sampling ranging from 3 s (averaging of SPIDER density estimations over 100 frames) down to 0.5 s (averaging of SPIDER density estimations over 15 frames). It shows that increasing time sampling for live-cell imaging super-resolution can provide enhancement of the spatial signal and allows following structural and morphological changes.

Careful analysis of Fig. 4b reveals that the mitochondrial membrane appears quite blurry and thickened. To clarify this, we would like to refer to Supplementary Fig. S4. The profiles for the average cell image obtained over 1,000 frames and for the different acquisition times (snapshots) taken at 3 s, 10 s, 20 s and 30 s with a 3 s time sampling (averaging over 100 frames) are compared. Changes in cell position and shapes can be noticed along the acquisition, which is typical behavior for dynamic imaging of live cells when the sample moves over a distance larger than the spatial resolution during data acquisition. However, we would like to point that dynamic super-resolution imaging with SPIDER allows following typical changes observed for live-cell imaging which brings that increasing time sampling not only leads to faster dynamics but also an enhancement of the spatial signal. In order to show this potency and compatibility of SPIDER with fast super-resolution experiments, we provide in Fig. 5 the SPIDER images that were obtained when reducing time sampling for different acquisition times. We demonstrate that we can reconstruct high-fidelity super-resolution images with a time sampling down to 0.5 s. This was performed by integrating of SPIDER images over only 15 frames on which, on average, 320 fluorophores were identified per frame. The dynamics of structures that would evolve, undergo remodeling, or fold into various shapes, could in this way be resolved. Finally, in order to estimate the resolution of the super-resolution images obtained, we report in Supplementary Fig. S5 the results obtained applying Fourier Ring Correlation (FRC)^3^. The results show that the resolution improves with an increasing sampling time. An estimated resolution of 55 nm was obtained for the SPIDER image obtained averaging over all frames whereas, over a time sampling of 0.5 s, the resolution was limited to 95 nm, in agreement with the apparent detail in the images provided in Supplementary Fig. S5.

## Conclusion

Live-cell imaging is required to investigate fast cellular dynamics and increasing efforts are currently made on the developments of methods to improve not only the spatial resolution but also the temporal resolution, or sampling time, of super-resolution imaging. We have shown that high-density sparse deconvolution of super-resolution images considering the implementation of an L_0_-norm provides more quantitative images, with reduced bias, better recall rate and higher accuracy. Overall, the proposed algorithm (SPIDER) works over a broad range of high-density images and allows detailed studies of dynamical cellular processes. It can be used to analyze high-density super-resolution data for fast imaging when investigating highly dynamic structural and morphological changes that mitochondria and other membrane organelles undergo to accommodate the continuous processes occurring in live cells.

## Methods

### The penalized regression model

Consider one image, indicated by ***Y***, that is a matrix of numbers representing the intensity of the pixels of the camera. This image is a blurred version of the latent image, a matrix ***X***, which consists of a number of point-like objects of varying intensities corresponding in super-resolution microscopy to the spatial distribution of active fluorophores. The blurring is described by a convolution with a fixed PSF. For every pixel in ***Y***, the size of the grid spacing in ***X*** is smaller: there are, say, 4 by 4 sub-pixels in ***X***for every camera pixel. Most of the elements of ***X*** will be zero, because there are no emitters located in the corresponding sub-pixel. Ignoring noise and other disturbances, a general linear relationship between ***X*** and ***Y*** is given by 
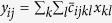
 where the array 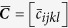 represents the PSF. It is a four-dimensional array with a special structure, reflecting the fact that the PSF is assumed not to vary with position. However, the details are not important for the model. As it is inconvenient to work with sums, we can exploit matrix-vector operations if we vectorize ***Y*** to ***y*** and ***X*** to ***x***, by stacking their columns on top of each other. Similarly, we can transform the array 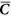 into the matrix ***C***, leading to ***y*** = ***Cx*** + ***e***, where we have introduced ***e***to represent the noise.

This description works from ***x*** to ***y***, but we are interested in the reverse problem: we want to obtain a good estimate of ***x***, given ***y***. This can be formulated as a least squares regression problem: find ***x*** that minimizes 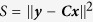, where 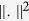 indicates the sum of the squares of the elements of a vector (also known as the L_2_-norm).

The number of unknown, the elements of ***x**,* is much larger than the number of observations, the elements of ***y***. It is well-known that in this case there is no unique solution to the regression problem. A popular and effective way out is to introduce a constraint on ***x***, a so-called penalty. A constraint that promotes sparsity is the sum of the absolute values of ***x***: 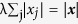. This penalty is called the L_1_-norm. It is added to the sum of squares: 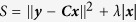. In the statistical literature, this approach is known as the LASSO (Least Absolute Shrinkage and Selection Operator^18^). The influence of the penalty is tuned by the parameter *λ*: the larger *λ*, the more sparse the estimation of ***x***, 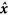.

The LASSO does a good job for image deconvolution, but our experience has shown that we can do better by switching to an L_0_-norm. The penalty becomes 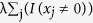 where 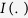 is the indicator function, which is 1 if the logical condition in its argument is true, and 0 otherwise. Symbolically we write 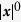. The objective function then becomes 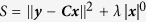. It has no explicit solution, but we can approximate it in an iterative way. We start reasoning from the obvious identity 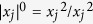. Suppose 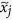 to be an approximation to 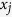, then we have that 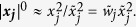 This gives us a weighted square and we can write 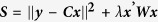, with the explicit solution 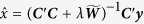 where an accent indicates the transpose of a matrix.

The idea is to repeatedly solve ***y = Cx***improving 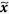 and hence 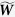 in each iteration. In practice, this works surprisingly well and only a handful of iterations are needed to do so. In fact, we use a slightly modified algorithm, taking 
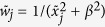
, with *β* a small number, to avoid instabilities when 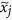 gets too close to zero. This modification also means that we never get 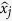 that are exactly zero.

The physics of the experimental situation prescribes that the elements of ***X*** are either zero or positive. We like the same to be true for our estimated 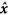. To this end, we use an asymmetric penalty on the size of the elements of **x**, which is large when they are negative and zero otherwise.

### Preprocessing

Smooth background intensity can occur in large parts of the observed image and it jeopardizes the sparsity of the estimated latent image. We have applied an approach^19^,^21^,^22^ that works well for image background estimation and correction of experimental super-resolution imaging data. It uses two-dimensional tensor products of splines and asymmetric penalized least-squares fitting to estimate the background (a spatial smooth surface). The tensor products are formed from two spline bases, which represent the splines in the row direction (first spatial direction) and the splines in the column direction (second spatial direction). Smoothness is tuned by penalizing the difference between adjacent spline coefficients and asymmetry is encoded incorporating an appropriate weight matrix in the least squares. For further details, we would like to refer to the references above.

It should be noted that preprocessing was only used on the experimental data, and not on the simulated images.

### Rendered images

Contrary to multiple-fluorophore localization methods, SPIDER does not return molecules coordinates but reconstructs a high-resolution image of molecule densities on a predefined sub-pixel grid whose size is defined by the oversampling factor *s*. The raw results that were obtained for the HEK293-T cell data (Figs 4 and 5 and Supplementary Fig. S4) have undergone a simple Gaussian rendering with a PSF the size of a pixel of the super-resolution grid (i.e. 25 nm) to generate super-resolution images.

### Software

We have implemented the algorithm in MATLAB using home-made routines. The code for analysis and a set of simulated and real example data are included in the Supplementary Software S1.

### Simulation maps

#### Photon model

The relative intensity of each of the fluorophores in each pixel was computed by numerically integrating a normal cumulative distribution function with a pre-defined Full-Width at Half Maximum (FWHM) over the area of the pixels. The simulated emitters can switch between a bright and a dark fluorescent state, which is described by a two-state model where k_on_ = 1/τ_off_ and k_off_ = 1/τ_on_ are the rate constants from the “on” to “off” and the “off” to “on” states, respectively. The activity of the fluorophores was modeled by a time-continuous Markov process of which the characteristic times were sampled from exponential distributions with the corresponding decay times. Throughout all the simulation maps, an average detected photon count per emitter per frame of 5,000 was taken, with a standard deviation of 2,000.

#### Noise statistics

To accommodate for shot noise, the number of photons measured in each pixel from a given frame was drawn from a Poisson distribution. Camera read-out noise was added in a second step and was assumed to follow a normal distribution with a variance of 1, centered on 0. For the simulation maps of the random images, a background of 100 photons per pixel was added. However, taking into account the TIRF mode used for the data acquisition, a background of 10 photons per pixel was added for the simulations of the geometric structures.

#### Random images

For the different simulations of images with randomly distributed emitters, *N* active molecules were randomly positioned on an area of 24 × 24 pixels. This area was placed in the middle of a 32 × 32 pixel grid in order to avoid a border effect during the evaluation of the performance of the algorithm. The width of the PSF was set to 390 nm. The size of the pixels was chosen to be 166 nm (or 100 nm for the second set of simulation images), resulting in a PSF to pixel size ratio of 2.35 (or 3.9, respectively).

For the simulations used in Fig. 1 and Supplementary Table S1, the image had a density of 1.25 μm^−2^ (i.e. 20 emitters on the 24 × 24 pixels map). For the sake of clarity, we simulated an ideal situation in Fig. 1 for which the position of each emitter was set at the middle of a pixel, with contributions from isolated, slightly overlapping and almost completely overlapping emitters. This ensures that the graphical results can be comprehended easily, as an illustration of the basic principle of the approach. On the other hand, for Supplementary Table S1, the position of each emitter was placed randomly on images (without matching pixel center).

#### Geometric structures

For the simulation of the geometric structure, molecules were placed on a 27 × 27 pixel grid (with a pixel size of 100 nm). The size of the pixels was chosen to match the standard deviation of the PSF. Three juxtaposed circles of diameter 400, 600 and 800 nm were simulated of which the fluorophores were positioned regularly every 20 nm on each circle over 300 frames. An average of 18 active fluorophores per frame was simulated (corresponding to a τ_off_/τ_on_ ratio of 31). This lead to strongly overlapping emissions and a molecular density estimated to be approximately 15 μm^−2^.

### Method evaluation

The overall performance and reliability of the different image analysis algorithms applied can be defined by several benchmark metrics. The first is recall rate, which describes the overlap between the set of detected fluorophores and the set of true positions and thus, it evaluates the number of simulated positions that can be correctly identified from the analysis of a single image. The second benchmark metric is accuracy, which evaluates the average distance between the positions of the true fluorophores and the detected ones. In addition to the recall rate and the accuracy metrics, we also report the number of false positives. This translates to the number of emitters that were found but cannot be associated to any true emitter. Lastly, we evaluated sparsity of the solutions obtained, i.e. the total number of non-zero pixels found by the algorithm divided by the number of fluorophores actually present.

However, due to the fact that the positions of the true fluorophores on the simulation maps are placed randomly, it is possible that multiple non-zero pixels (detected fluorophores) can be associated to them on the super-resolved image. For this reason, a ‘virtual’ fluorophore is used and its position is calculated from the center of mass of the group of pixels assigned to the same true fluorophore (see Supplementary Fig. S1 and reference^13^). A prerequisite for a detected fluorophore to contribute to the estimation of a virtual fluorophore is that a true fluorophore can be found within a pre-defined lateral tolerance disk (of which the diameter was 200 nm in all the results). When multiple true fluorophores are present within the lateral tolerance disk of a detected fluorophore, this latter gets assigned to the closest one. If no true fluorophore can be found within the lateral tolerance disk of a detected fluorophore, it is considered to be unidentified and thus a false positive.

### Sample preparation

HEK293-T cells were cultured in DMEM medium supplemented with 10% (v/v) FBS, 1% (v/v) glutaMAXTM and 0.1% (v/v) gentamicin (all Gibco^®^, ThermoFisher Scientific, Aalst, Belgium) grown at 37 °C and 5% CO_2_ and subcultured every 3–4 days. One day before transfection, ~250,000 cells were seeded in a 35 mm glass bottom dish No. 1.5 (MatTek, Ashland MA, USA) and incubated overnight. Right before transfection, cells were washed 3 times with 1.5 mL PBS (pH 7.4, Gibco^®^, ThermoFisher Scientific, Aalst, Belgium) and new, preheated medium was added. Cells were transfected using a calcium phosphate method. 32 mL CaCl_2_ (2 M) solution was mixed with 5 μg plasmid DNA (pcDNA3:DAKAP-Dronpa)^20^ in 218 μL ultrapure water. This mixture was added dropwise to 250 μL 2 × HBS (50 mM HEPES, 280 mM NaCl, 1.5 mM Na_2_HPO_4_ at pH 7.05–7.06, filter sterilized) and immediately added to the cells. Approximately 8 hours post transfection, the cells were washed 3 times with PBS (pH 7.4) and supplied with fresh growth medium. Imaging was performed 24–36 hours post transfection. Before imaging, the growth medium was removed from the cells and replaced with 2 mL HBSS.

### Optical imaging

Imaging experiments were performed on an Olympus cellTIRF microscope described elsewhere^23^ equipped with an additional EMCCD camera (ImagEMX2, Hamamatsu). Image sequences of 1,000 frames were recorded with constant 488 nm laser excitation and a camera exposure time of 30 ms. The direct EM gain was varied between 50 and 900 to accommodate for the differences in fluorescence output. Data files were exported as tiff files without any further software processing.

## Additional Information

**How to cite this article**: Hugelier, S. *et al.* Sparse deconvolution of high-density super-resolution images. *Sci. Rep.* **6**, 21413; doi: 10.1038/srep21413 (2016).

## Supplementary Material

Supplementary Information

Supplementary Video S1

Supplementary Video S2
