# Corrigendum: Sparse deconvolution of high-density super-resolution images

**DOI:** 10.1038/srep24975

**Published:** 2016-05-09

**Authors:** Siewert Hugelier, Johan J. de Rooi, Romain Bernex, Sam Duwé, Olivier Devos, Michel Sliwa, Peter Dedecker, Paul H. C. Eilers, Cyril Ruckebusch

Scientific Reports 6: Article number: 21413;10.1038/srep21413 Published online 02252016; Updated: 05092016

A marked-up version of the Supplementary Information was inadvertently published with the original version of this Article. In addition, Supplementary Software 1 was omitted. These errors have now been corrected.

