# Supplementary material for: Sparse deconvolution of high-density super-resolution images
Source: Sci Rep. 2016 Feb 25;6:21413. doi: 10.1038/srep21413 (PMC4766479; doi:10.1038/srep21413)
Supplement: Supplementary Information [file srep21413-s1.pdf]

Sparse deconvolution of high-density super-resolution images (SPIDER)

Siewert Hugelier[1], Johan J. de Rooi[2], Romain Bernex[1], Sam Duwé[3], Olivier Devos[1], Michel Sliwa[1], Peter Dedecker[3], Paul H. C. Eilers[2], Cyril Ruckebusch[1]

[1.] Université de Lille, LASIR CNRS UMR 8516, F-59000 Lille, France;

[2.] Erasmus MC, Department of Biostatistics, Rotterdam, the Netherlands;

[3.] Department of Chemistry, KU Leuven, Belgium.

Correspondence should be addressed to C.R. (cyril.ruckebusch@univ-lille1.fr).

**Supplementary File**	**Title**
**Supplementary Notes**	Computational strategies for SPIDER
**Supplementary Table S1**	Comparing the results obtained with the L_0-norm penalty and the L_1-norm penalty in super-resolution imaging
**Supplementary Table S2**	Calculation time of deconvolution methods
**Supplementary Table S3**	Figures of merit for a set of emitters with a random placement at different densities
**Supplementary Figure S1**	Method for performance evaluation
**Supplementary Figure S2**	Performance benchmark of CSSTORM, FALCON and SPIDER for a PSF to pixel size ratio of 3.9
**Supplementary Figure S3**	Insights on the data and results of a series of three juxtaposed circles
**Supplementary Figure S4**	Fast SPIDER super-resolution for mitochondria in a HEK293-T cell labeled with DAKAP-Dronpa
**Supplementary Figure S5**	Estimation of the image resolution by Fourier Ring Correlation (FRC) on the HEK293-T cell labeled with DAKAP-Dronpa
**Supplementary Video S1**	Raw movie data for a HEK293-T cell
**Supplementary Video S2**	SPIDER on an image patch showing a clear recovery of individual emitters
**Supplementary Software S1**	SPIDER algorithm

Supplementary Notes. Computational strategies for SPIDER

Theory

The observed image ***Y*** can easily be more than 10^5 pixels and thus gives a vector ***y*** with more than a hundred thousand elements. The vector ***x*** is much larger: with a sub-grid oversampling of 4 by 4, it will have 1.6 million elements and the matrix ***C*** will have that many rows and columns. Although it is sparse, it might not fit into memory and even if it did, computation times would be huge.

Fortunately, we do not have to solve the whole system at once: we can split ***Y*** into sub-images (patches) and do the regression for each of them. Suppose ***Y*** has n by n pixels and the oversampling factor is s. Then ***X*** will have $m = sn$ rows and columns and the matrix ***C*** will have $m^2 = s^2n^2$. Solving a system of p equations takes time proportional to p^3, so for the full system the time needed would be proportional to s^6n^6. If we divide ***Y*** into k by k patches, we need a time proportional to $s^6(n/k)^6$ for each of them. There are k^2 patches, so total time would be proportional to s^6n^6/k^4. This is an attractive result: dividing **Y** in 4 by 4 patches already speeds up the calculations 256 times. This analysis shows the principle of divide-and-conquer, but it ignores one important aspect. A proper model for an observed patch of ***Y*** should include a border area at all sides with half the width of the PSF, to account for contributions by emitters at the borders. We call this width b. This means that a patch has dimensions $n = k + 2b$ by $n = k + 2b$ and thus at a certain value of k, the contribution of b gets so influential that there will be no gain anymore. We also observed that in any iteration to find the L_0 penalized solution, elements of $\tilde{\boldsymbol{x}}_j$ that are close to zero stay close to zero. So they can be eliminated from the equations beforehand, giving an enormous speed-up. More elements are made zero as a result of the iteration, so we can eliminate more and more of them in the subsequent ones.

Practice

The SPIDER algorithm is, as already said, sensitive to high oversampling of the image patches as the matrices used for calculation can be too large to fit the memory of the computer. Therefore, we have performed a test on the exact times SPIDER needs to perform a calculation (per frame) for different patch sizes (k = 4, 8, 16 and 32 pixels) at different oversampling factors (s = 1, 3, 5 and 7) with 50 repetitions per calculation. Calculations were also performed for CSSTORM. The results are shown in **Supplementary Table S2**. The data set used for the calculations are the 50 random simulation maps with a density of 10 μm^{-2}. One should keep in mind that the density of the data set also plays a role in computation time (more emitters on the map mean more signal and thus a more complicated fit). The overlap (i.e. border area b) between the different patches, to accommodate for emitters on the border of the patches, was 4 pixels on every side of the patch.

The calculations were performed on a computer with an Intel(R) Core(TM) i7-4770 CPU @ 3.40GHz CPU and 16 GB RAM memory. The computer works with a 64-bit Windows 7 professional operating system. The version of Matlab used for these calculations is the R2014a version.

Penalized least-squares with an L_0-norm		
**Oversampling factor (*s*)**	**False Positives (-)**	**# emitters (-)**
1	0	46
2	0	48
3	0	36
4	0	35
5	0	30
6	0	32
7	0	26
8	0	22
**Penalized least-squares with an L_1-norm**		
**Oversampling factor (*s*)**	**False Positives (-)**	**# emitters (-)**
1	16	77
2	8	86
3	7	84
4	6	76
5	5	75
6	9	61
7	9	56
8	7	57

Supplementary Table S1. Comparing the results obtained with the L_0-norm penalty to the ones obtained with the L_1-norm penalty in super-resolution imaging. The results obtained on a simulated map of 20 randomly distributed emitters, which corresponds to a molecule density of 1.25 μm^{-2}, are reported. All the solutions obtained represent the sparsest solutions with a recall rate of 100 %. The analysis was done with an oversampling factor *s* increasing from 1 to 8, therefore decreasing the size of the pixels on the super-resolution grid from 166 nm to 21 nm. The simulated emitters are located at random positions on the images. One can notice that the L_0-norm penalty always provides sparse solutions without false positive emitters (for a lateral tolerance disk with diameter 200 nm). This makes SPIDER an excellent method for quantitative analysis of real data.

SPIDER (in seconds)				
Patch size (*k*)	Oversampling factor (*s*)			
	1	3	5	7
4	0.145 ± 0.015	3.938 ± 0.120	17.874 ± 0.603	60.689 ± 2.694
8	0.097 ± 0.020	2.356 ± 0.088	12.095 ± 0.485	45.280 ± 2.418
16	0.169 ± 0.019	2.055 ± 0.063	11.946 ± 0.515	MEM
32	0.187 ± 0.017	2.663 ± 0.106	148.630 ± 34.210	MEM

CSSTORM (in seconds)				
Patch size (*k*)	Oversampling factor (*s*)			
	1	3	5	7
4	79.212 ± 8.369	80.916 ± 3.443	93.811 ± 2.239	139.428 ± 5.722
8	21.860 ± 1.023	37.321 ± 1.457	135.103 ± 2.753	419.323 ± 7.414
16	10.363 ± 0.555	213.276 ± 6.468	*	*
32	147.898 ± 6.121	*	*	*

Supplementary Table S2. Calculation time of deconvolution methods. Calculation times (in seconds) for SPIDER and CSSTORM on the 50 random simulation maps of density 10 μm^{-2}. The calculations are done for different patch sizes (*k* = 4, 8, 16 and 32 pixels) and different oversampling factors (*s* = 1, 3, 5 and 7). The border area *b* was taken to be 4 pixels on every side of the patch. MEM: insufficient memory for calculations; *: calculation times > 1800 s. It should be clear that SPIDER is faster than CSSTORM with a factor 10 – 100.

Figures of merit for randomly positioned fluorophores

Density (μm^{-2})	Recall (%)	Accuracy (nm)	False positives (-)
0,5	5,33 ± 7,78	128,09 ± 47,89	7,55 ± 0,65
2	19,24 ± 6,59	125,13 ± 21,35	25,07 ± 2,24
4	31,08 ± 6,50	117,76 ± 11,55	39,30 ± 3,95
7	41,75 ± 6,86	109,52 ± 6,99	48,16 ± 5,46
9	46,27 ± 7,20	104,93 ± 6,07	48,37 ± 6,11
12	50,54 ± 7,61	99,15 ± 4,74	45,22 ± 6,78
15	52,95 ± 7,82	94,33 ± 3,94	39,53 ± 6,73

Supplementary Table S3. Figures of merit for a set of emitters with a random placement at different densities. The table reports the figures of merit obtained by using emitters with a random placement as 'found' emitters. The figures of merit were obtained by using 2,500 combinations for every density, for a lateral tolerance disk with diameter 200 nm. The number of emitters with random placement used for the evaluation was equal to the number of true fluorophores (i.e. sparsity of 100 %).

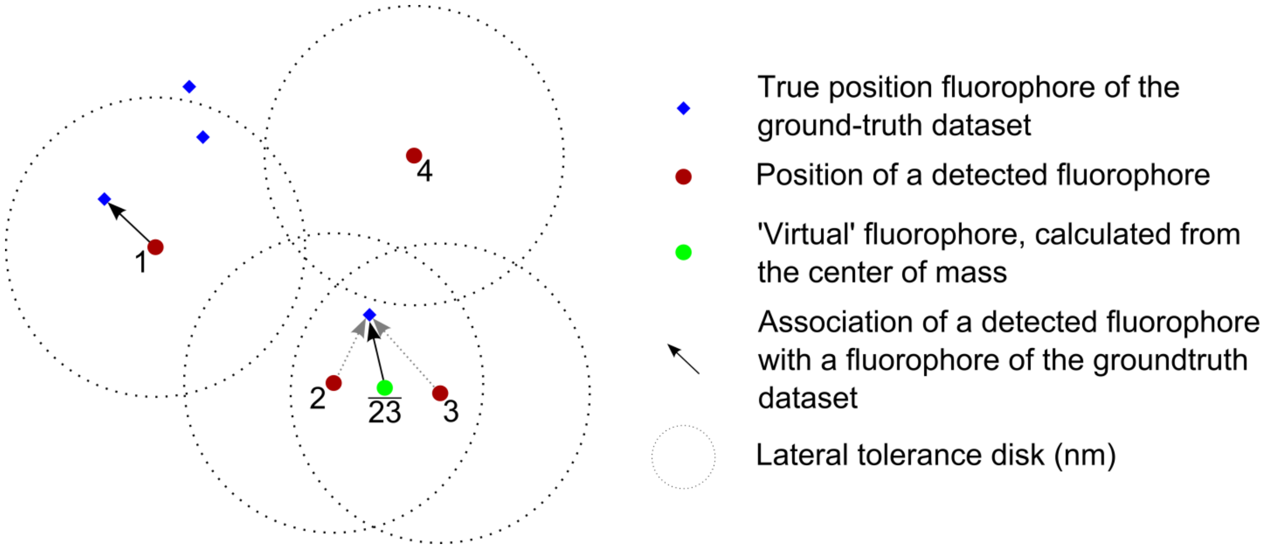

Supplementary Figure S1. Method for performance evaluation. Graphical explanation of the method evaluation, representing the four different figures of merit (recall, accuracy, false positives and sparsity) that are used. In this figure, one can recognize the true positions of the fluorophores of the ground-truth dataset in blue and the positions of the detected fluorophores in red. For every detected fluorophore, a lateral tolerance disk is drawn around it to visualize to which true fluorophores it can possibly be assigned. When it can be assigned to multiple true fluorophores, it will be assigned to the closest one. If a true fluorophore is assigned to multiple detected fluorophores, a 'virtual' fluorophore is calculated from the center of mass of these detected fluorophores and further calculations are done using this 'virtual' fluorophore. This example has a recall of 50 % (2 of the 4 fluorophores of the ground-truth dataset detected), an accuracy which is the average of length of the black arrows, 1 false positive (fluorophore 4) and a sparsity of 100 % (4 detected fluorophores for 4 true fluorophores).

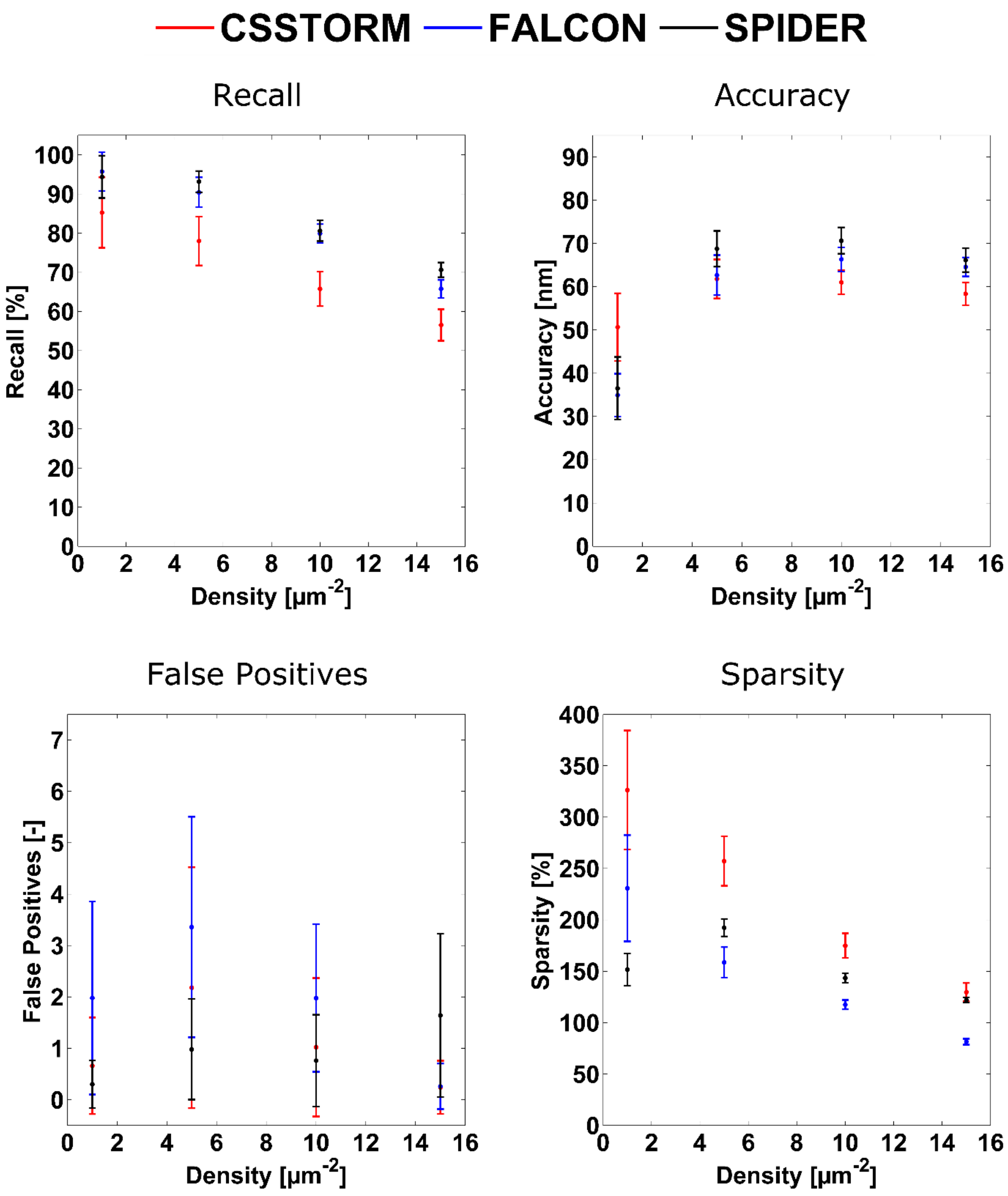

Supplementary Figure S2. Performance benchmark of CSSTORM, FALCON and SPIDER for a PSF / pixel size ratio of 3.9. The simulation images correspond to an average photon number of 5,000 per molecule (standard deviation of 2,000) and a background of 100 photons with a Poisson distribution. The comparison shows that FALCON (blue) performs better than the results shown in **Figure 2** of the manuscript (PSF / pixel size ratio of 2.35), as expected for a localization-based method, whereas SPIDER performs equally well. This shows that SPIDER performs steadily in different situations with different PSF / pixel size ratios.

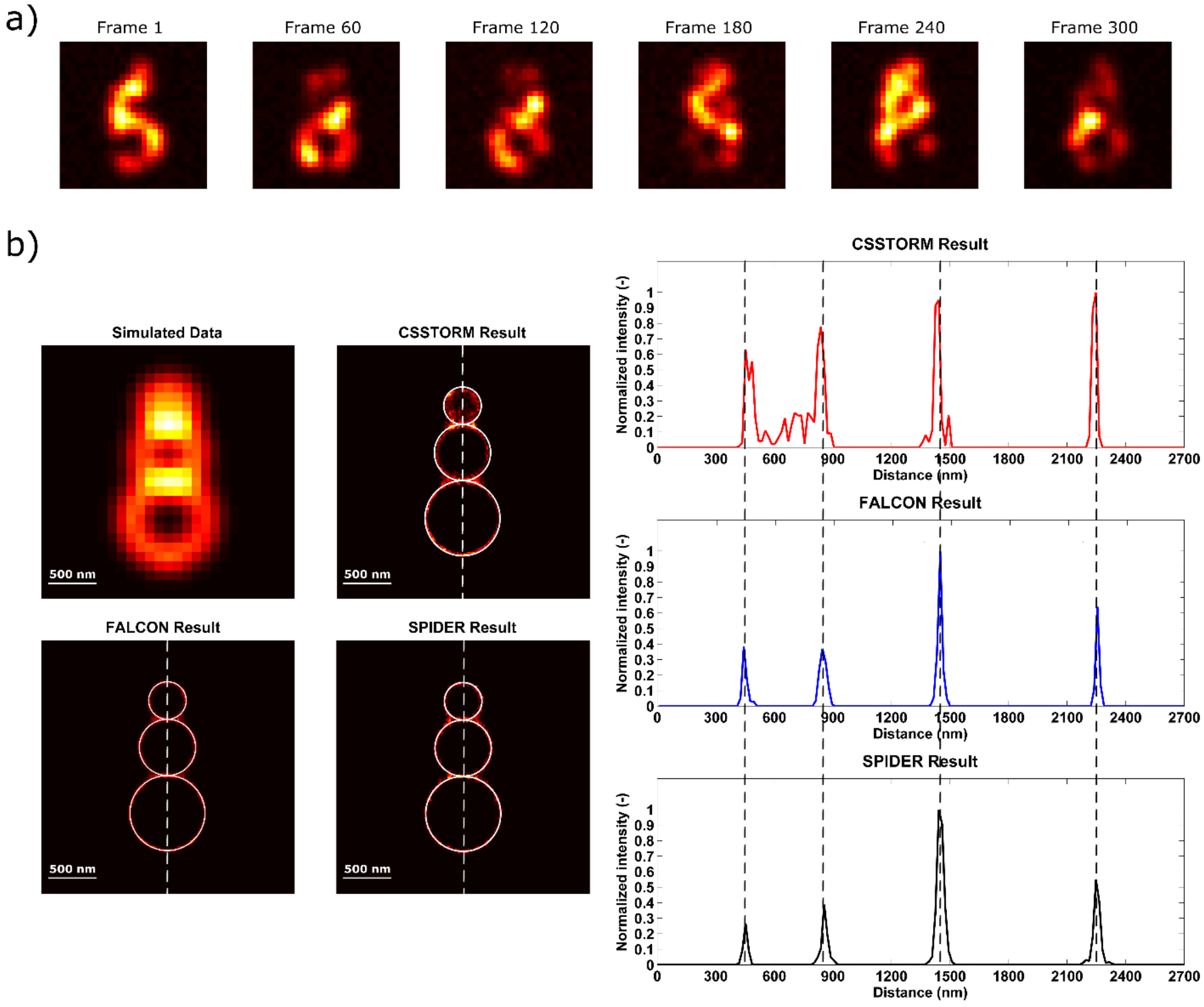

Supplementary Figure S3. Insights on the data and results of a series of three juxtaposed circles. We report in (a) several frames composing the simulated data of the three juxtaposed circles of size 400, 600 and 800 nm and in (b) the longitudinal cross-sections of the indicated line for CSSTORM, FALCON and SPIDER. The different frames presented in (a) show the high density of the data and the huge overlap of emitters (τ_{off}/τ_{on} ratio of 31, leading to a fluorophore density of 15 μm^{-2} per frame). The longitudinal cross-sections of the three juxtaposed circles then show that CSSTORM gives biased results, towards the middle of the simulated circles, while FALCON and SPIDER allow the structure to be reliably recovered.

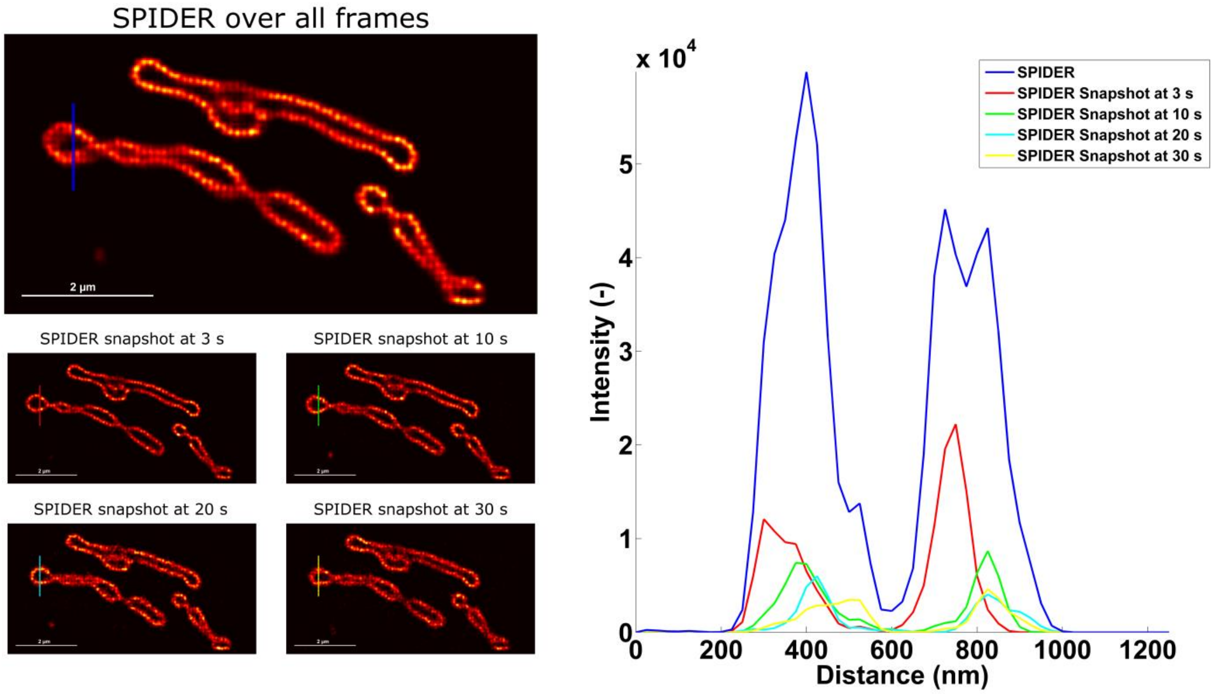

Supplementary Figure S4. Fast SPIDER super-resolution for mitochondria in a HEK293-T cell labeled with DAKAP-Dronpa. The live-cell images of a region sized 4 µm by 8 µm (region indicated by the box in **Fig. 4a** of the manuscript) obtained averaging over 1,000 frames (blue) and different snapshots at times 3 s (red), 10 s (green), 20 s (cyan) and 30 s (yellow) with a time sampling of 3 s are shown in the maps. The profiles, corresponding to the lines marked in these images, are shown in the graph next to it. They show that averaging the results obtained over all the frames leads to a loss of resolution. It should be clear that the blurred and thickened membrane, obtained by averaging the signal over all the frames, is actually due to a movement of the cell along the acquisition as the center of the membrane changes along the profiles of the different snapshots.

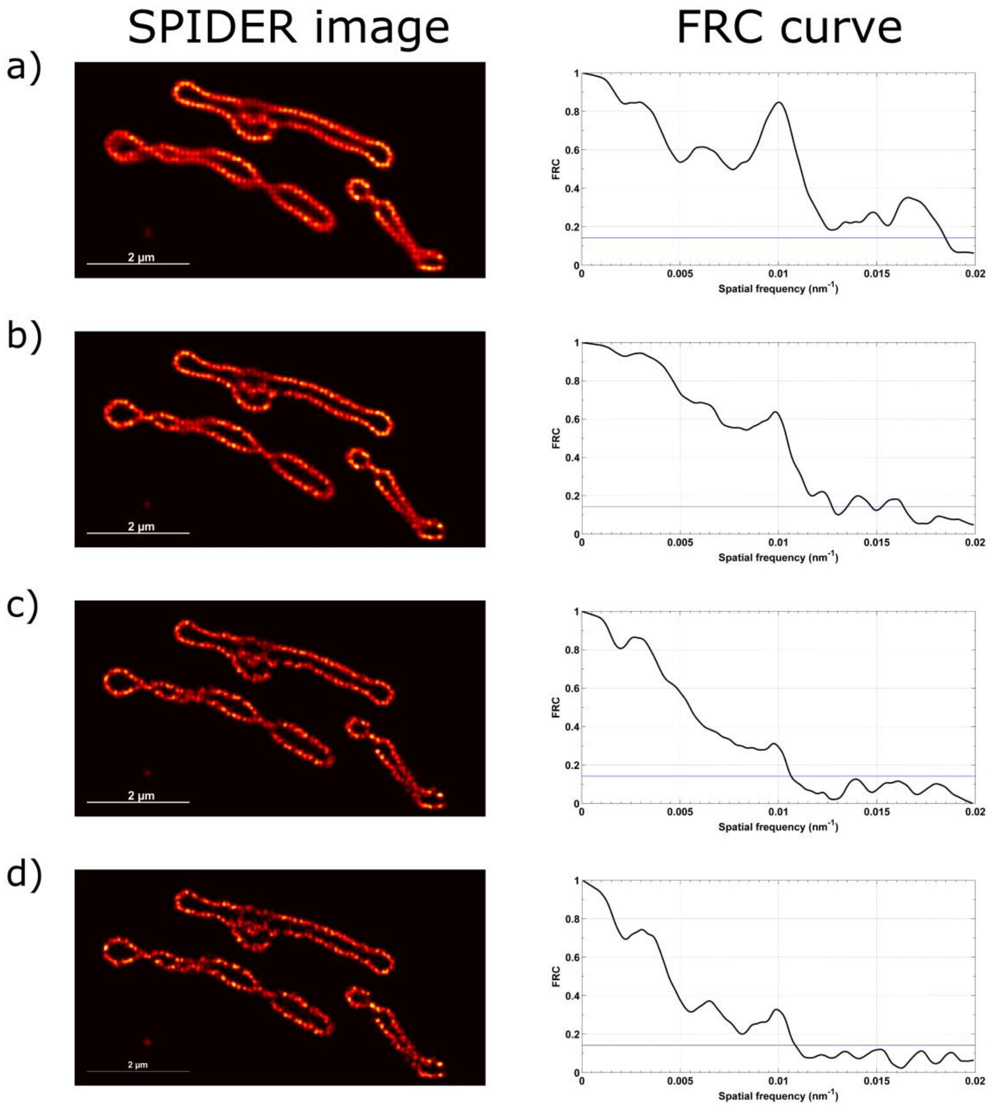

Supplementary Figure S5. Estimation of the image resolution by Fourier Ring Correlation (FRC) on the HEK293-T cell labeled with DAKAP-Dronpa. The image resolution has been estimated on the results obtained by SPIDER. The figure shows the SPIDER image (rendered with a 25 nm PSF) and the corresponding FRC curve (in black) of the resulting image over (a) all frames; (b) the first 3 seconds (100 frames); (c) the first second (30 frames) and (d) the first 500 ms (15 frames). Respective values for image resolution are 54.45 nm, 78.98 nm, 94.78 nm and 92.73 nm. The threshold value (shown in blue) used here is 1/7, a value corresponding to to the literature for localization microscopy images (see reference 3 in the manuscript). The FRC curves translate that image resolution improves with increasing sampling time. However, the effects of sample shifting (see **Supplementary Fig. S4**) and sub-sampling on a super-resolution grid are observed as well, both decreasing when sampling time decreases.

Supplementary Video S1. Raw movie data for a HEK293-T cell

Movie that shows the acquired data (frame per frame, 30 fps) for mitochondria in HEK293-T cell. The movie (512 x 512 pixels, 100 nm pixel size) is reconstructed from 1,000 camera frames (2 dummy frames, not shown) with an acquisition time of 30 ms per frame. It shows the high density of the acquired data. Emitters can never be observed independently (in every individual frame), which leads to a high degree of overlap at all times. This results in few observable blinking. The wide-field image (mean image of the 1,000 frames) and the resolved SPIDER image (mean image of the 1,000 super-resolution frames) can be found in **Figure 4a** in the manuscript. Scale bar: 10 µm.

Supplementary Video S2. SPIDER on an image patch showing a clear recovery of individual emitters

This movie (40 x 80 pixels, 100 nm pixel size) is a collection of individual frames obtained by the SPIDER super-resolution method for mitochondria in HEK293-T cell, shown in **Figure 3** of the manuscript. It shows the individual spotted positions, frame per frame, for 100 frames (corresponding to 3 s of data acquisition) for the highlighted zone. Scale bar: 1 µm.

Supplementary Code S1. Spider algorithm

The supplementary code includes all the functions needed to perform the SPIDER analysis on high-density images. It uses a simple interface routine that serves as a buffer between the user and the calculations and is easily adaptable to the needs of these high-density images. Two demo sets (one on simulated data and one on experimental data) have been included in order to demonstrate the algorithm.
